# Indirect Estimation of Vertical Ground Reaction Force from a Body-Mounted INS/GPS Using Machine Learning

**DOI:** 10.3390/s21041553

**Published:** 2021-02-23

**Authors:** Dharmendra Sharma, Pavel Davidson, Philipp Müller, Robert Piché

**Affiliations:** 1 VTT Technical Research Centre of Finland, Kaitoväylä 1, 90570 Oulu, Finland; dharmendra.sharma@vtt.fi; 2 Faculty of Information Technology and Communication Sciences, Tampere University, 33720 Tampere, Finland; pavel.davidson@pp.inet.fi (P.D.); robert.piche@tuni.fi (R.P.)

**Keywords:** gait analysis, ground reaction force, ground contact time, INS/GPS, machine learning, deep neural network

## Abstract

Vertical ground reaction force (vGRF) can be measured by force plates or instrumented treadmills, but their application is limited to indoor environments. Insoles remove this restriction but suffer from low durability (several hundred hours). Therefore, interest in the indirect estimation of vGRF using inertial measurement units and machine learning techniques has increased. This paper presents a methodology for indirectly estimating vGRF and other features used in gait analysis from measurements of a wearable GPS-aided inertial navigation system (INS/GPS) device. A set of 27 features was extracted from the INS/GPS data. Feature analysis showed that six of these features suffice to provide precise estimates of 11 different gait parameters. Bagged ensembles of regression trees were then trained and used for predicting gait parameters for a dataset from the test subject from whom the training data were collected and for a dataset from a subject for whom no training data were available. The prediction accuracies for the latter were significantly worse than for the first subject but still sufficiently good. *K*-nearest neighbor (*K*NN) and long short-term memory (LSTM) neural networks were then used for predicting vGRF and ground contact times. The *K*NN yielded a lower normalized root mean square error than the neural network for vGRF predictions but cannot detect new patterns in force curves.

## 1. Introduction

**Figure 1:**
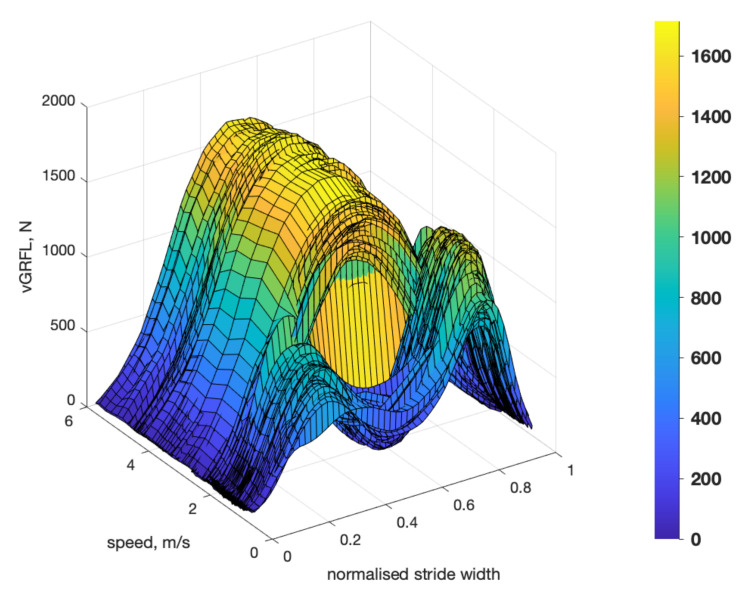
Example of vertical ground reaction force of left foot with respect to speed and normalized stride duration.

Contemporary approaches for gait analysis include direct measurement of boundary conditions (ground reaction forces *GRF* and moments *GRM* or alternatively the center of plantar pressure *CoP*) under each foot [1]. Currently, stationary, floor-mounted force plates or instrumented treadmills are being used as gold standard setup to measure these parameters. Typical vertical GRF (vGRF) curves during walking and running are shown in Figure 1.

Alternatively, gait parameters can be measured by instrumented insoles [2]. Although current pressure insole sensors show limited durability and high sensitivity to their boundary condition in the shoe, this technology has been shown to achieve competitive average accuracy for measuring vGRF [3]. Some important parameters like the ground contact time can be computed from the GRF data.

Indirect estimation of ground reaction forces and ground contact time (GCT) using inertial measurement units (IMU) is a growing field of research thanks to the development of small wearable sensors and machine learning techniques (see [4] for a comprehensive review). The benefits of using IMUs to compute GRF and GCT are cost effectiveness, ease of use, and accessibility to the general population [4]. Usually these methods use either regressions, random forests, artificial neural networks, or convolutional neural networks to compute relationships between the acceleration vector and gait features [5,6,7,8,9,10]. However, due to the physiological differences between people, these methods require calibration or training data for each person to estimate gait features with reasonable accuracy [11,12].

One drawback of most proposed methods is that they require to model the biomechanical system to a certain extent [1]. This modeling requires extensive knowledge of subject-specific parameters (e.g., mass, body dimensions, moment of inertia, etc.), which causes inaccuracies and uncertainty [4]. For example, in [13] the authors approximate the GRF with an artificial neural network and measurements from a single IMU, which has to be located on a subject’s center of mass.

Other challenges in the estimation of ground reaction forces include the determination of antero-posterior and medio-lateral components and GRF in situations where both feet touch the ground, and the estimation of peak forces [4]. Therefore, in the literature the focus is usually on estimating only vertical ground reaction forces and ignore antero-posterior and medio-lateral components. In this manuscript, the restriction to the estimation of vGRF is due to the insoles used as reference, as they only measure the vertical component. We solve the challenge of estimating GRF when both feet touch the ground using a deep neural network.

In [12], we presented a measurement setup for continuous 3D analysis of gait mechanics and technique during outdoor running and walking on level terrain, which was based on an inertial navigation system (INS) combined with a Global Positioning System (GPS) receiver (INS/GPS) and a gait segmentation algorithm. The aim of this paper is to present a methodology for indirect estimation of vGRF, GCT, and some other target features that can be directly measured only by instrumented insoles or by force plates. Parameters such as peak or average vGRF of single steps suffice for many applications and can be computed also on consumer-grade devices. We estimate the parameters using traditional machine learning techniques (bagged ensemble trees) and compare them to measurements and target feature values derived from instrumented insoles. In addition, we use *K*-nearest neighbors (*K*NN) and deep neural networks for estimating vGRF and GCT label curves.

## 2. Materials and Methods

**Figure 2:**
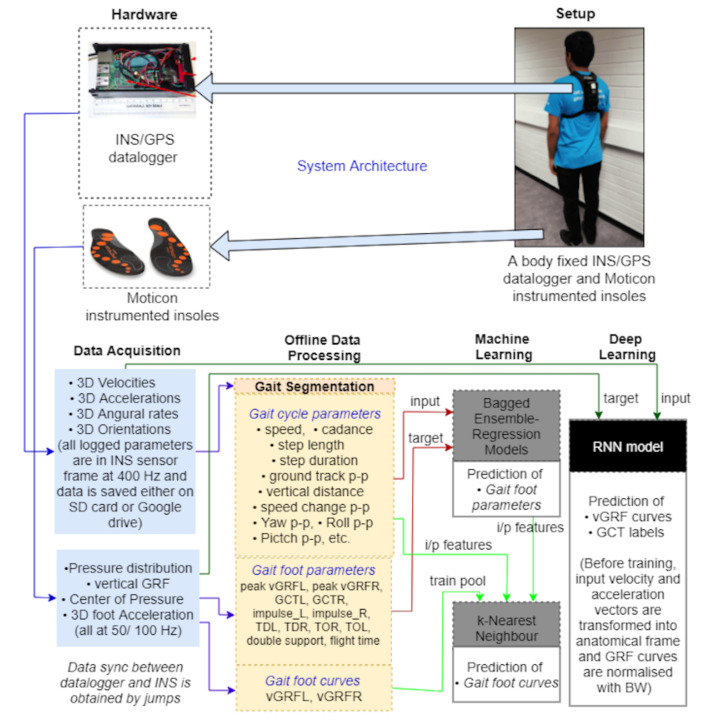
Measurement setup and the sequence of data processing steps used in this study.

This section describes how to predict the vertical component of GRF and GCT using a single body-mounted INS/GPS device, instrumented insoles, and different supervised machine learning approaches. Figure 2 shows the measurement equipment and data processing workflow. The INS/GPS datalogger has been explained in [12]. Moticon instrumented insoles [14] were used for training, validation and providing reference for GRF and GCT.

### 2.1. Measurement Setup

The measurement setup consisted of an INS/GPS datalogger and instrumented insoles. The datalogger included a Raspberry Pi-3 model B board (Raspberry Pi Foundation, Cambridge, UK) running Linux OS, a Vectornav VN-200 GPS-aided inertial navigation system (INS/GPS), a GPS antenna, and a 4200 mAh power bank (see Figure 2). INS/GPS data were collected on a memory card in the datalogger’s Raspberry Pi. The INS/GPS data were sent to the memory card through a wired connection, which ensured that no data were lost. After the experiment, the data were transmitted to cloud storage using a 4G/LTE USB modem connected to the Raspberry Pi. Data from the Moticon insoles were collected on the internal memory of the insoles. Jumps with both feet were executed at the beginning and at the end of data recording, to obtain simultaneous toe-off and touch-down times for both feet. The jumps caused sharp peaks in the vGRF measured by the insoles and the vertical velocity measured by the datalogger. Using these peaks, it was possible to better synchronize the insole data of both feet and insole data with datalogger data. The synchronization of insole and datalogger data as well as the data analysis was done offline on a computer after the experiment. A detailed description of this setup was provided in [12]. For our outdoor walking and running tests on a level track, the datalogger unit was attached to the torso of the test subject. The idea was to use the datalogger in a similar way as commercially available heart rate monitor straps. Therefore, we refrained from considering noise sources that are caused, for example, by backpack rotation or movement if it is not fastened tightly on the back. A risk for constant misalignment of the datalogger remains but is uncritical as the IMU can align itself and output data were computed in the geographical coordinate frame.

The Moticon wireless sensor insoles measured the pressure from 13 capacitive pressure sensors per insole, which cover approximately 50% of the insole surface [14]. Pressures between 0.0 and 40.0 N/cm${}^{2}$ can be recorded with a resolution of 1.0 N/cm${}^{2}$. The accuracy in peak total force measurements in walking is claimed to be ±25% [15]. In [3], OpenGo insoles were tested for various movements. The force impulses measured by OpenGo insoles were 13–34% below those of the force plates, but very highly correlate to the measurements recorded by force plates (0.8–1 in most situations). For the tests in this paper pressures were sampled at 50 Hz. The Moticon insoles were fitted inside Asics-DS-trainer-16 neutral running shoes by replacing the original insoles.

### 2.2. Data Acquisition

The INS/GPS data were computed in the geographical coordinate frame. Position, velocity, acceleration, orientation, angular velocity, and ground track (the path on the Earth’s surface) were measured at a sampling rate of 400 Hz [12]. Most known studies on walking and running biomechanics use IMUs with output rates of up to 100 Hz. However, this is not enough to track body dynamics during sprint running when the step frequency is about 5 steps per second [16]. Our field experiments confirmed that the selected output rate of 400 Hz satisfies demands of running biomechanics.

With the Raspberry Pi-3 short delays can occur and a synchronization error of up to 2.5 ms is possible, which is less than the used sampling rate. Thus, even for fast running the synchronization error is unproblematic.

The VectorNav VN-200 INS/GPS sensor underwent a robust calibration and acceptance testing process at the manufacturing facility. According to VectorNav, the INS/GPS yields velocities with an accuracy of ±0.05 m/s in real-time application. Its inertial heading accuracy is 0.3° root mean square (RMS), while for pitch and roll the RMS is 0.1°. The angular resolution is less than 0.05° and the repeatability less than 0.1°.

In order to achieve the claimed accuracy, a good GPS signal without multipath is required. It could be improved by postprocessing [17]. In order to obtain accurate heading estimates the subject had to move at a speed greater than approximately 1.5–2 m/s, which is slightly above the preferred walking speed. At standstill the heading accuracy dropped to approximately 1°–2°, depending on the magnetic environment [12].

The insoles logged pressures for each of the 13 sensors in an insole and 3D accelerations using an accelerometer in the insoles, and computed total pressure, plantar pressure distribution, and two centers of pressures. However, in this paper only vertical foot force and pressure were used (see [12,18] for details). One aim of our study was to develop algorithms that enable insoles to be replaced with the datalogger without significant accuracy degradation of vGRF estimates.

### 2.3. Data Processing

The required data processing has been described in detail in [12]. Accelerations and velocities were computed in the anatomical frame. Strides were segmented using our gait segmentation approach from [12]. Once the data were segmented into steps, features or metrics that are commonly used in walking and running were computed for each step to facilitate analysis of the data (see [12,18] for details). The following features are of importance in the analysis in the later sections of this paper:

- Step length: distance traveled during one step
- Vertical displacement: peak-to-peak difference in vertical movement
- Speed averaged over one step: arithmetic mean of the speed (=step length/duration of step)
- Contact time: stance phase duration
- Double support time (only during walking): time when both feet touch the ground
- Flight time (only during running): time when both feet are in the air

**Figure 3:**
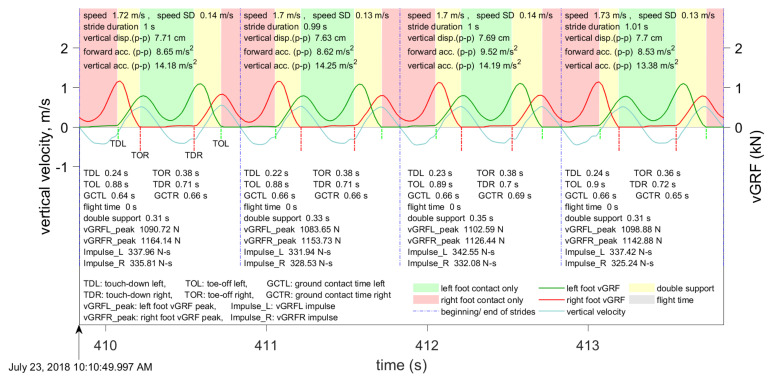
Example of the data computed by the INS/GPS and Moticon insoles during walking.

**Figure 4:**
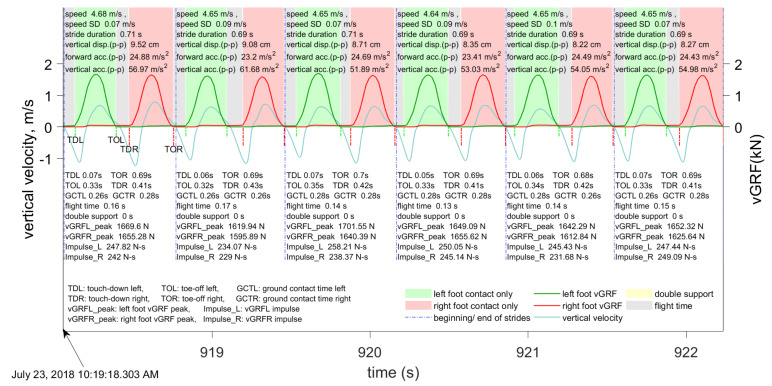
Example of the data computed by the INS/GPS and Moticon insoles during running.

Examples of segmented data and features computed for each stride in walking and running are shown in Figure 3 and Figure 4, respectively. Speed and its standard deviation (speed SD), stride duration, vertical displacement, touch-down and toe-off times, and forward and vertical accelerations were obtained from the INS/GPS. Peak vGRFs, impulses, ground contact times, flight times, and double support times were obtained from the insoles.

### 2.4. Feature Engineering

The purpose of feature engineering is to identify valid, useful, and understandable patterns in INS/GPS data that have strong correlation with the target parameters but minimum inter-correlation with the other features. The target parameters list includes parameters that can only be extracted from insoles, namely, GRF curve, peak vGRF, impulse, ground contact time, flight time, and double support time.

In machine learning, optimal feature selection is crucial for developing simple yet reliable prediction models. Of equal importance are strong absolute correlations between input and target features. In addition, the input features chosen for training prediction models should be uncorrelated or only weakly positively or negatively correlated to each other to avoid accuracy degradation when applied to unseen (test) data [18].

It is well known that speed is negatively correlated with GCT and double support time (see, e.g., in [19]); speed is also positively correlated with flight time, peak vGRF, and impulse. Therefore, speed is considered as one input feature.

Even if the datalogger is tightly mounted on the test subject’s back, there is a small chance of shifting. Furthermore, the sensor orientation for different subjects generally differs. Therefore, features derived from 3D angle were ignored, because the angle oscillations depend on the orientation of the VN-200. The 3D angular velocities are also orientation dependent, since they are logged in the sensor frame, and were also ignored.

**Figure 5:**
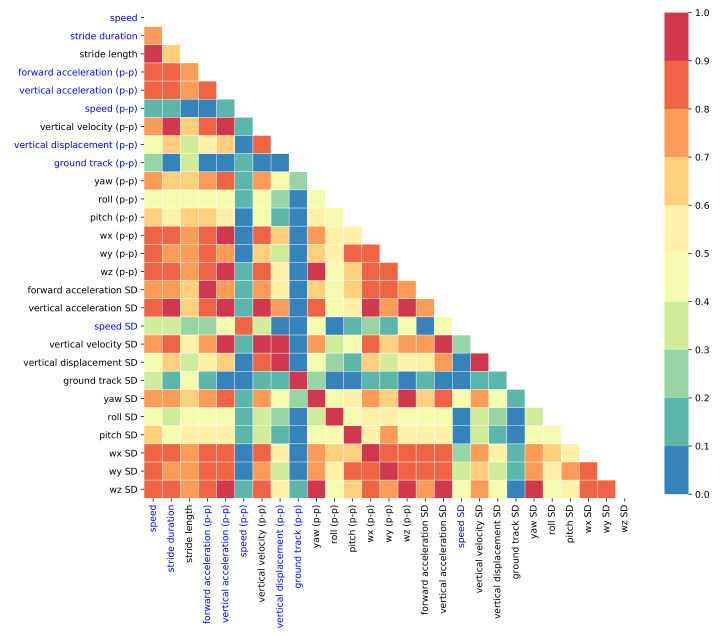
Absolute correlations between input features. Optimal input features in blue.

Figure 5 shows the absolute correlations between pairs of input features. The additive (p-p) means that the range of the input feature is used, e.g., speed (p-p) is the difference between minimum and maximum speed of stride data vectors; the additive SD indicates that the standard deviation of stride data vectors is used as feature. The standard deviation-based input features are highly correlated (absolute correlations > 0.9) with the corresponding range (p-p) input features, except for speed. Therefore, they can be eliminated/ignored.

**Figure 6:**
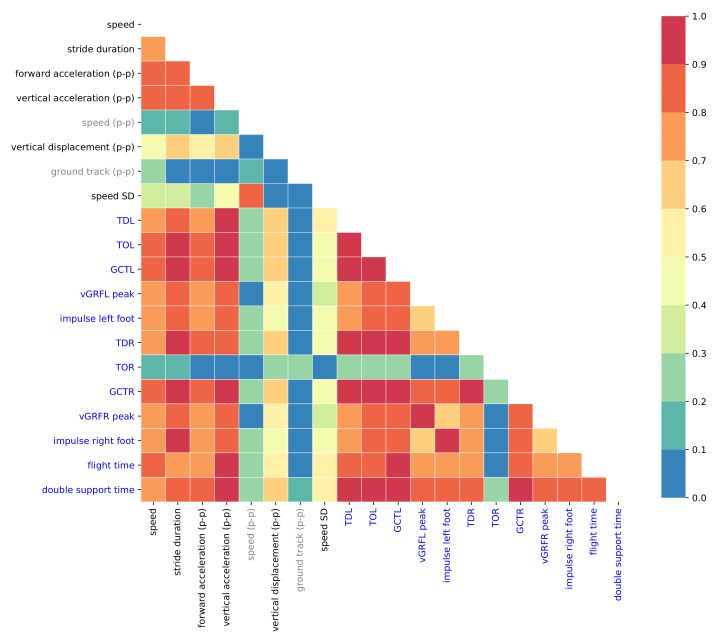
Absolute correlations between optimal input and target features (in blue).

The optimal input features were selected by correlation-based feature selection. If the absolute cross-correlation between any two input features was larger than 0.9 then one of them was dropped. The set of optimal input features consisted of speed, stride duration, forward acceleration (p-p), vertical acceleration (p-p), speed (p-p), vertical displacement (p-p), ground track (p-p), and standard deviation of the speed vector. Figure 6 shows the absolute correlations between the eight optimal input features and the target features, which are the gait parameters.

Figure 6 shows that speed (p-p) and ground track (p-p) are only weakly correlated (≤0.3) to any of the target values. Therefore, they too can be omitted from the list of optimal input features, which now consists of only six input features: speed, stride duration, forward acceleration (p-p), vertical acceleration (p-p), vertical displacement (p-p), and standard deviation of speed.

Furthermore, the toe-off time of the right foot (TOR) is only weakly correlated (≤0.3) to the input features. Prediction results for TOR were inaccurate, which is in line with the weak correlations. Thus, TOR was omitted and finally 11 target features were retained: Touch-down time of left foot (TDL), toe-off time of left foot (TOL), ground contact time of left foot (GCTL), peak vGRF of left foot (peak vGRFL), impulse left-foot, touch-down time of right foot (TDR), ground contact time of right foot (GCTR), peak vGRF of right foot (peak vGRFR), and impulse right-foot.

### 2.5. Traditional Machine Learning Techniques

The Matlab regression learner app was used to train regression models for the remaining 11 target foot gait parameters. Furthermore, flight time and double support time can be predicted with sufficient accuracy, although they depend on the movement of both feet. The regression models of peak vGRFs and impulse (for both feet) were trained after normalizing the target parameters using the subject’s body weight, in order to make the prediction models independent of a subject’s weight (see [18] for details).

Three types of regression models were trained for the 11 target parameters:

- Bagged ensemble of regression trees (min. leaf size: 8 with 30 learners) by considering six optimal features as input (referred to as 6 features in the remainder of this paper).
- Bagged ensemble of regression trees (min. leaf size: 8 with 30 learners) by considering principal component analysis ([20], p. 260 f.) (PCA) with six numeric components.
- Bagged ensemble of regression trees (min. leaf size: 8 with 30 learners) by considering all 27 features as input (referred to as all features).

Furthermore, the *K* Nearest Neighbor (*K*NN) approach (see in [21], p. 174 ff.) for details) was used for selecting strides from a training dataset that would match peak vGRF and time parameters of test strides. The results are presented in Section 3.3.

### 2.6. Deep Neural Networks

The vGRF curve can be split into single strides, which each have two distinct characteristics, height (peak vGRF) and width (stride duration, GCT, toe-off (TO), and touch-down (TD)) [18]. The challenge is that the stride characteristics are continuously changing during locomotion. Traditional machine learning-based methods for predicting the stride characteristics are highly dependent on the size of a training set containing characteristics from various strides. Furthermore, they are limited to detecting only known strides and cannot detect new patterns in the force curve.

Deep neural networks do not suffer from these two major drawbacks and enable the prediction of each point in the vGRF curve using current and previous raw measurements of INS/GPS logged data. Furthermore, the deep learning method enables the estimation of GCT labels, with 1 indicating that the foot is in contact with the ground and 0 indicating that the foot is in the air. The predicted vGRF and GCT (label) curves can then be compared to the vGRF measured by the Moticon insoles and the GCT labels that are derived by postprocessing the vGRF and foot pressure data from the insoles.

One approach for the estimation of vGRF and GCT by neural networks has been discussed in [12]. As the aim is to predict continuous vGRF and GCT curves the estimation problem is a time series problem, which means that a recurrent neural network (RNN) is a potential solution. Because current vGRF and GCT depend on large numbers of previous data points, long short-term memory (LSTM) or gated recurrent unit (GRU) RNN architectures are believed to yield better predictions than vanilla RNN [12]. In this paper, two separate LSTM neural network regression models were trained for vGRF and GCT predictions.

## 3. Results

This section explains the collected data. In addition, it presents and discusses the results for vGRF feature predictions using the methods explained in Section 2.5 and the results for vGRF and GCT curve predictions using the methods explained in Section 2.6.

### 3.1. Data Description

In this paper, data from the INS/GPS datalogger and Moticon insoles for two healthy adults were used (subject 1: male, 72 kg, 178 cm, 26 years; subject 2: male, 64 kg, 171 cm, 27 years). The INS/GPS data were synchronized manually with Moticon insole data. After gait segmentation using the approach described in [12], three different datasets were prepared.

**Figure 7:**
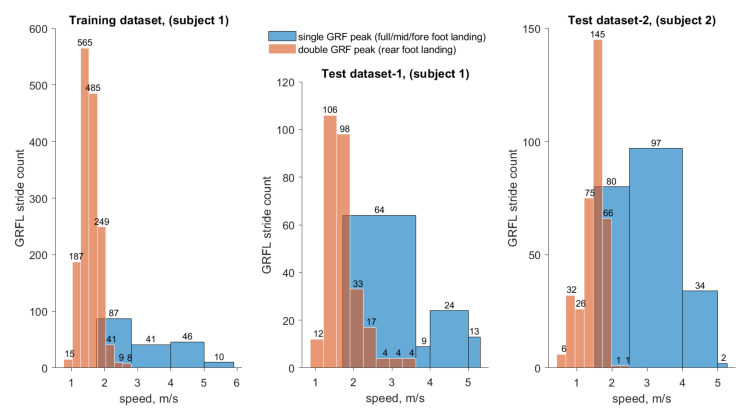
Speed-stride histograms for training set and both test sets.

All three datasets were extracted from continuous motion data for a walk-run experiment with varying speeds. Therefore, each dataset contained strides from walking, speed-walking, jogging and running. The training dataset consisted of 1743 strides from subject 1. The mean stride duration was 1.039 s, and the speed varied between 0.77 and 5.90 m/s. INS/GPS data were measured at 400 Hz resulting in 725,017 data points. Two test datasets were prepared: one containing 388 strides from subject 1 and one containing 565 strides from subject 2. The speed distribution (foot landing) of chosen strides is displayed in Figure 7. Double GRF peak indicates data from walking or speed-walking while single GRF peak indicates data from jogging or running. In walking, a foot touches the ground with the heel first. Then, the weight is transferred to the forefoot before toe off. Thus, there are always two peaks. In running, only the forefoot touches the ground. While the speed for strides from jogging and running, in general, is higher than the speed for strides from walking and speed-walking, Figure 7 reveals that there is no particular speed threshold that could be used to decide whether the force curve should take the shape of a “M” (i.e., two force peaks in a stride) or an inverted “V” (i.e., one force peak in a stride). The analysis of the strides showed, furthermore, that the stride curve also depended on the type of foot landing. This was clearly noticed for speed-walking and jogging.

As can be seen from Figure 7, the training data consists of mostly walking strides (1559 strides, 89.44% of all strides) of medium walking speed. Only 184 strides from running are included in the training data. For the two test sets the amount of walking and running strides are significantly closer: test set 1 contains 278 walking (71.65% of all strides) and 110 running strides and test set 2 contains 352 walking (62.30% of all strides) and 213 running strides.

### 3.2. vGRF Feature Prediction Results

The three types of regression models described in Section 2.5 were trained for each of the 11 foot parameters using segmented data of the training dataset. Foot parameters for both test sets were then estimated from data collected with the INS/GPS datalogger and compared with the Moticon insole measurements.

Because some of the parameters, such as peak vGRFs (vGRFL_peak and vGRFR_peak) and foot impulses (impulse_L and impulse_R), depend on the subject’s bodyweight [18], the regression models were trained with target data normalized by the bodyweight (BW; i.e., divided by BW). This helped to estimate the target parameters of another subject with different body weight. The regression models were used to predict normalized target parameters which were then multiplied by the body weight of the subject to obtain target parameter estimates.

**Figure 8:**
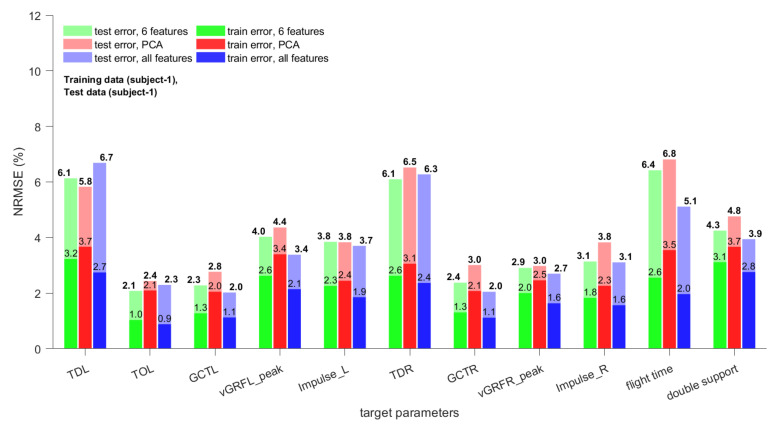
Normalized root mean square errors of three types of regression models (see Section 2.5 for details) for gait parameter estimates of training set and test set 1.

**Figure 9:**
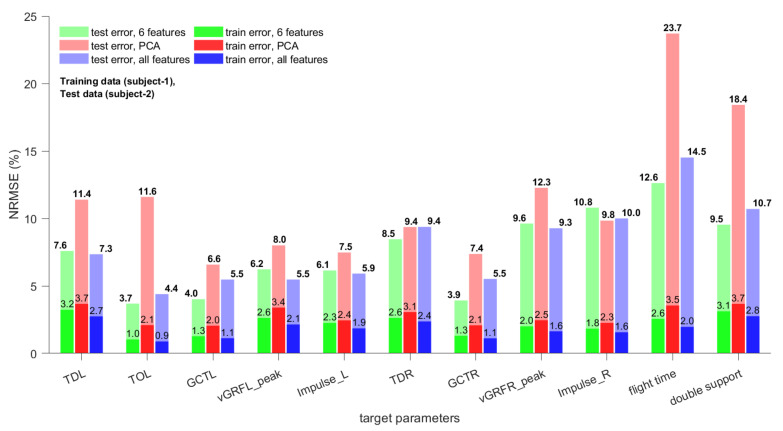
Normalized root-mean square errors of three types of regression models for gait parameter estimates of training set and test set 2.

The normalized root mean square errors (NRMSE) values for all 11 gait parameters based on the three types of regression models for the test sets of subject 1 and subject 2 are shown in Figure 8 and Figure 9, respectively. Both subjects differed in body weight and height, which makes it likely that also their gait characteristics differ. The NRMSE values in Figure 8 and Figure 9 support this assumption; the errors for test set 1 were, in general, significantly lower than those for test set 2. As the training data were from subject 1 there should be no difference in gait characteristics for the training set and test set 1.

A decision whether an achieved NRMSE is sufficient or not depends on various factors, such as the application. Based on the feedback received from experts, athletes, and coaches, the achieved accuracies for training set and test set 1 are sufficient for walking and endurance running. For sprint running (by professional athletes) and “the last few steps in jumping events” the accuracy might be insufficient. As our interest was to develop a datalogger and estimation methods that could be used for walking and (endurance) running applications, the feedback was encouraging and confirmed our assumption. For example, the vertical GRF varies usually between 1.0 and 1.5 BW for walking and 2.0 and 2.9 BW for running [22]. Thus, NRMSEs of 2.7–4.4% are sufficient. For example, for a vGRF of 2.5 times the BW this corresponds to average errors between 0.0675 and 0.1100 BW. Test set 2 contained strides from subject 2, and its errors were sometimes 3–4 times as high as those of the training set. This means that predicting gait parameters for a subject for which no training strides are available significantly increases the NRMSEs of the estimates. While the accuracy of force related parameters is still acceptable, the NRMSEs for time related parameters might be too high for certain applications.

It is important to note that the TD and TO parameters depend on the stride segmentation method as they are events and their time is measured with respect to the beginning of the gait strides. GCT, peak vGRF, and foot impulse depend on the complete stride vector and should be less dependent on the stride segmentation method [18].

The regression models using either the six optimal features or all 27 features as input yielded the lowest training errors (models trained on six optimal features provided lowest NRMSE for nine of 22 parameters; models trained on all 27 features provided lowest NRMSE for 12 of 22 parameters; for Impulse_R of test set 1 both provide smallest NRMSE). Only for TDL of test set 1 and Impulse_R of test set 2 the regression model trained on the first six principal components yielded the lowest NRMSE. However, the other two regression models performed equally. Based on these results, the use of either all features or the six optimal features as input for the ensemble bagged trees can be recommended as they usually yield similar NRMSEs. In order to make a decision which of the input sets should be chosen more data, preferably from different subjects, should be collected and analyzed.

### 3.3. vGRF Curve Prediction Approach and Results (*K*NN)

In order to construct the vGRF curve for a test set, it is essential to select vGRF strides from the training data that match both peak vGRF and time parameters (stride duration, GCT, TO, and TD) of the test strides. A simple approach for picking a target stride is the *K*-Nearest Neighbor (*K*NN) approach (see in [21], p. 174 ff. for details). It can be used to find a stride from the set of training strides that best matches the test stride with respect to the time parameters. The peak vGRF is not considered here, because of its different scale. Like the bagged ensemble trees, the *K*NN works with segmented data.

First, the vGRFL and vGRFR training strides normalized by body weight were divided into separate collections for left and right foot strides. Then, their respective foot time parameters were either calculated or estimated by the bagged ensemble of regression trees models based on the six optimal features. For left foot vGRFL strides, the input feature space included stride duration, TOL and GCTL. While stride duration could be directly calculated from the data, TOL and GCTL had to be estimated by the regression models. Due to the large NRMSE of TDL predictions, this parameter was omitted. Similarly, for right foot vGRFR strides, stride duration and predicted GCTR were considered as input feature space (TOR was omitted from the set of target features. See Section 2.4 for details.). The respective foot time parameters were then used for calculating the Euclidean distance between test stride and training strides. Using the Euclidean distance as similarity measure, the stride indices of the *K* most similar vGRFL and vGRFR training strides were sought and the values were averaged to obtain vGRF stride predictions. Our analysis revealed the optimal *K* to be between 5 and 12. For these values the results varied insignificantly. The NRMSE for the first test dataset (subject 1) for continuous curve generation were 6.63% for vGRFL and 5.37% for vGRFR with K=8 neighbors.

### 3.4. Predictions by Deep Neural Network

Separate LSTM neural network regression models for vGRF and GCT predictions were trained using the Keras frontend to Google’s TensorFlow. The training data consisted of six input feature vectors, including unprocessed 3D acceleration and 3D angular rates, and two target output feature vectors. Orientation quaternion, ground speed, and vertical velocity were ignored as the leave-one-out analysis in [12] revealed that they have no significant impact on the classification accuracy. In case of vGRF curve prediction, the target output feature vectors were vGRFL and vGRFR; in case of GCT label predictions, the target output feature vectors were GCTL-labels and GCTR-labels.

Feature selection was done based on the *consider only one* and *leave-one-out* approaches. It showed that forward and vertical acceleration were the most important input vectors, being highly dependent, to predict the vGRF and GCT labels. Adding lateral acceleration and 3D angular velocity improved the predictions to some extent. Adding further input features did not improve the prediction accuracies.

**Figure 10:**
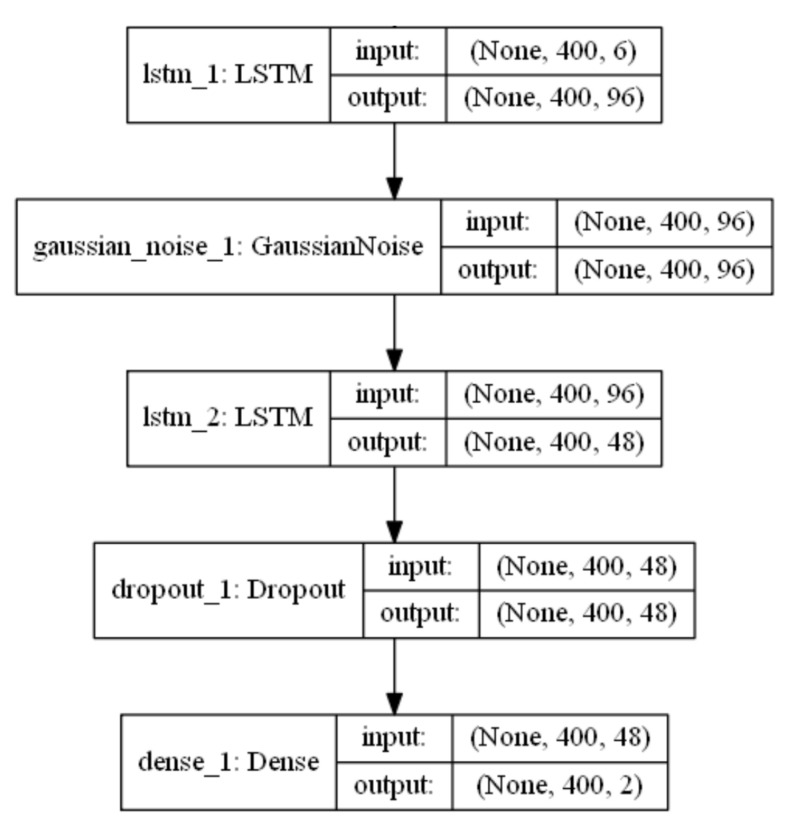
Structure of the long short-term memory (LSTM) neural network regression models used for predicting vertical ground reaction forces (vGRF) and ground contact time (GCT).

In order to find the best neural networks, several combinations of LSTM and GRU layers were tried by considering different numbers of neurons in layers and by changing the hyperparameters. The tests showed that the performance of the neural network is robust against changes in number of neurons as long as it is ensured that the model can learn sufficient features of the input data. The used neural network model structure is shown in Figure 10.

The used NN had two LSTM layers along with one input noise layer in between, which improved the network robustness towards test data noise. The noise layer applied additive zero-mean Gaussian noise to intentionally corrupt the input data to mitigate model overfitting and was only activated during the model training phase. After the second LSTM layer, a dropout layer was added to improve generalization by randomly dropping a specified ratio (dropout rate) of neurons. These dropped neurons had no effect on the activation on the neurons in the following layers during the training phase. The dropout layer was also activated for model training only. Finally, a fully connected dense layer was added to map the hidden layer data to the two independent target outputs.

The input data vectors, which were measured at 400 Hz, were scaled to values between −1 and 1 before being split into sequences of 400 samples with a shift of one sample for each new sequence. The reason for choosing sequences of 400 samples was the average stride duration of 1.039 s in the training data, which equals approximately 400 samples per stride. This choice yielded 724,616 training sequences for both input and target data. The first 399 samples of each sequence were history data and the last sample was the present input. Therefore, when calculating the loss function the initial 399 samples of the two estimated target sequences were ignored. Furthermore, no predictions for the first 399 example points in training and test datasets could be made.

Both the vGRF NN and the GCT label prediction NN shared the same model structure but used different activation functions. The GCT label prediction was a binary classification problem. Therefore, the sigmoid activation function was chosen at the output layer. For the fully connected dense layer in the vGRF model, the rectified linear unit (ReLU) activation function was used. In addition, both models used the *adam* optimizer with *binary_crossentropy* loss function for the GCT label model and *mean_squared_error* loss function for the vGRF model. After training these models, the point to point GCT labels and vGRF could be predicted without any direct foot pressure measurements from insoles.

**Figure 11:**
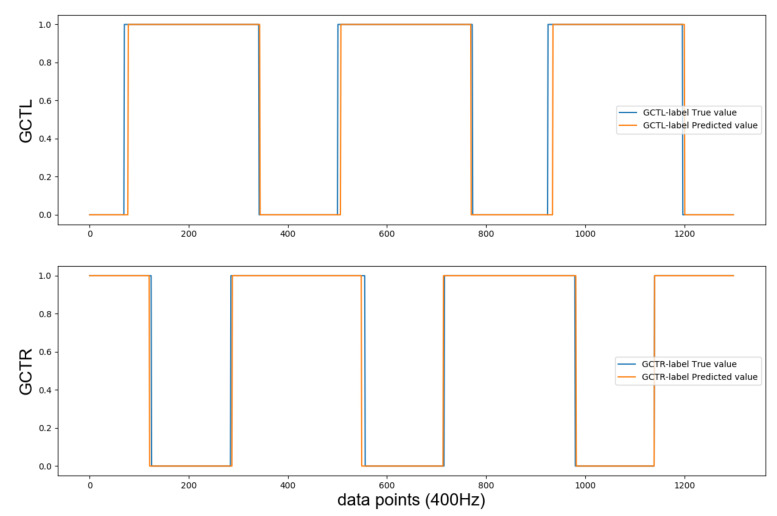
Glimpse of GCT label predictions for test set 1 (binary classifications obtained by applying threshold 0.5 to binary label probabilities).

The GCT label network yielded probabilities for the binary classes (1 = foot touches the ground; 0 = foot in the air), taking values in [0,1]. These probabilities were transformed into binary values by setting all values below 0.5 to 0 and all others to 1. Figure 11 shows a glimpse of the final binary label predictions. The accuracy for left and right foot binary GCT label predictions were 92.65% and 91.23%, respectively. Most prediction errors occurred during the TO and TD events, when foot transition was ongoing.

**Figure 12:**
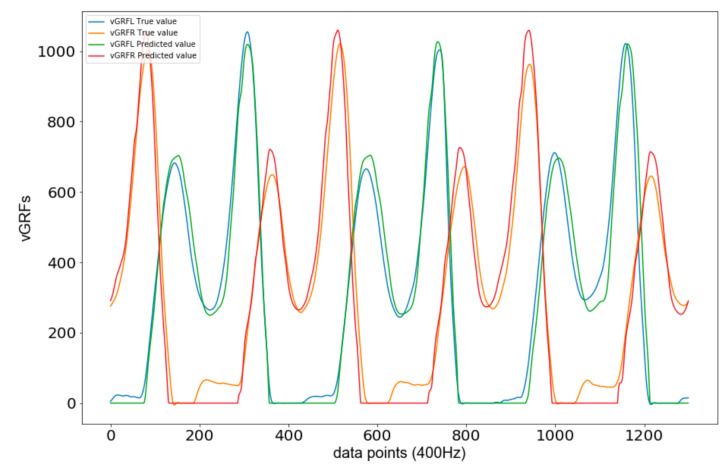
Glimpse of vGRF predictions generated by the LSTM neural network for test set 1.

Figure 12 shows a glimpse of the predicted vGRFL and vGRFR curves for test dataset 1. The curves accurately approximated the true vGRF measurements. The NRMSE in vGRF prediction were 8.38% for left foot and 8.54% for right foot, respectively. However, a closer look at the curves in Figure 12 shows that most of the significant errors occurred during the time when the foot did not touch the ground. In these phases the GRF is zero, as predicted by the neural network, yet the insoles recorded nonzero forces in the second half of these phases. The reason is that in the second half of phases without ground contact the foot moves back towards the ground, and towards the insoles (In the first half of phases without ground contact the foot moves upwards and away from the insoles.). Thus, toes and/or heels create some pressure on the insoles, which can be interpreted as noise. Therefore, the NRMSEs overestimate the actual errors.

## 4. Discussion

This paper presented a methodology for indirect estimation of vGRF and GCT using machine learning techniques and a wearable INS/GPS datalogger. For many applications it is unimportant to know the whole vGRF curve; it suffices to describe each stride by just a few parameters, such as peak or average vGFR during the stride and/or the ground contact time. For example, for musculoskeletal injury analysis and prevention, active and passive peaks of the vGRF are of great interest [9]. These parameters can be estimated by traditional machine learning techniques, which can be run on consumer grade devices. The advantages of the presented datalogger are that it is more cost effective and easier to use than floor mounted force plates or pressure measuring insoles. Furthermore, it uses only one sensor (location), thus removing the need for sensor synchronization, which is one crucial problem for the estimation of GRF from kinematic data [4]. The location of the datalogger on the upper back means that it does not move with respect to the body, which removes the need to model inertial properties [4].

In a first step, correlations between 27 features of segmented INS/GPS data were calculated, which reduced the list of relevant features to 8. The correlation of these 8 features with 12 gait parameters that could directly be extracted only from insoles were then computed in a second step, which enabled the elimination of two further features of segmented INS/GPS data. The analysis also showed that no features from INS/GPS data showed high correlation to the toe-off time of the right foot, so this parameter was eliminated from the further analysis. These feature selection steps were of importance for developing simple but reliable prediction models. For example, the *K*NN algorithm, which was used for vGRF curve predictions in Section 3.3, has a tendency to be fooled by irrelevant features, which can be avoided by using the set of six optimal features from the correlation analysis.

The 11 remaining foot gait parameters were then estimated from the features of segmented INS/GPS data using three types of bagged ensembles of regression trees and compared to the parameter values provided by the insoles. For the regression models the impact of feature selection was inconclusive; for some gait parameters models, using all 27 features yielded better estimates than models using the six optimal features and vice versa. This analysis, however, showed that using the regression models trained with the PCA-transformed data of the six optimal features generally resulted in higher NRMSEs. Furthermore, the impact of training data on the estimates was demonstrated. Gait parameter estimates in the test set for the subject whose training data were used for training the regression models had significantly lower NRMSEs than the estimates in the test set for the subject for whom no training data were available.

Peak vGRFs and foot impulses depend on an individual’s weight. Therefore, these parameters were normalized for body weight. For the vGRFs and foot impulses of the left foot this resulted in ≈50% higher NRMSEs for the subject without training data, which is promising. However, for the vGRFs and foot impulses of the right foot, the NRMSEs were more than 200% larger than those of the subject for which training data were available. The reason for this discrepancy is unclear, and further studies with more test subjects are planned to investigate this phenomenon and to obtain models that generalize better.

The final part of the analysis dealt with the predictions of vGRF and GCT label curves based on the INS/GPS data. *K*NN and a LSTM neural network were used for predicting vGRFs. The *K*NN provided lower NRMSEs for the test set of the subject from whom the training data were collected. It is, however, important to note that the performance of the *K*NN depends on the quality of the strides in the training set. Only if the training set contains strides that are similar to the strides that should be predicted the *K* can provide accurate estimates. This requires a large number of diverse strides in the training set, which in turn gives rise to the second main disadvantage of the *K*NN; its prediction time is linear to the number of samples in the training database [23]. This drawback can be somewhat mitigated by replacing the exhaustive search used in this paper for finding the *K* closest training strides by *k*-dimensional tree search [24].

The analysis showed that the LSTM neural network yielded vGRF estimates that were zero whenever the foot was not touching the ground. The insoles, whose vGRF measurements are used as reference in this paper, however, recorded nonzero forces when the foot moved downwards. Therefore, the accuracy of the predictions yielded by the LSTM neural network is even better than the errors given in this paper, which can be interpreted as an upper boundary.

To sum up, the regression models are used for estimating a small set of parameters for each stride, which suffices for many applications. The KNN yields vGRF curves based on the parameter estimates from the regression model and is therefore useful for more sophisticated applications. However, it is restricted to modeling strides as averages over training strides, meaning that it might not generalize well and that its curves can show non-zero vGRF while the foot does not touch the ground. The deep neural network is computationally demanding but yields vGRF and GCT curves with a frequency of 400 Hz. An advantage of the vGRF curves yielded by the deep neural network is that it correctly estimates vGRF to be zero when a foot does not touch the ground.

Comparing the achieved accuracies with those of related works is difficult, because the used equipment, conditions, and experiments themselves varied between different studies. However, the results from the literature can indicate whether our proposed system is competitive. For example, in [25] two different MLP network configurations were used for estimating vGRF during walking. The networks achieved NRMSEs of 4.7% and 4.8%, respectively. Guo et al. [11] proposed a proxy measurement approach and used sensors attached to the subject’s forehead, seventh cervical vertebra, and fifth lumbar vertebra. For walking data, the authors achieved NRMSEs between 3.8% and 4.2%. Jiang et al. [9] used four IMUs attached to foot, shank, distal thigh, and proximal thigh. A random forest regression model was used for estimating vGRF during walking and achieved NRMSEs of 1.70–2.33% depending on the sensor location. Our datalogger was attached to the subject’s upper back, which means that it did not move with respect to the body. For the peak vGRF we achieved NRMSE as low as 1.6% for training data and 2.7% for test data. The vGRF curves estimated by a *K*NN and a LSTM neural network yielded NRMSEs between 5.37% and 8.54%. Keeping in mind that we estimated vGRF during walking and running with the latter making the prediction more challenging, our combination of hardware and software is competitive and easy to use.

Furthermore, it is crucial to understand that the limiting factor is the accuracy of the insole measurements, as these measurements were used for training the models. Stöggl and Martiner found that force impulses recorded by the insoles were 13–34% below those recorded by a force plate system [3]. Thus, if training data from force plates would be used the accuracy of the estimates is believed to improve. The analysis in Section 3.4 showed that the insoles falsely measured non-zero vGRF for downward foot movements while the LSTM neural network yielded correct zero vGRF estimates. This explains part of the errors in the vGRF curve estimation. It also illustrates one key factor for replacing the insoles by our datalogger.

The proposed system of equipment, which was explained in detail in [12], and algorithms has several advantages. First, it uses a single sensor tightly fastened to the torso of the test subject. This removes the need for synchronization of sensors and does not require a model of the biomechanical system or knowledge of inertial properties (see in [4] for details). Second, the INS/GPS sends its measurements through a wired connection to the Raspberry Pi. In the literature, often sensor networks are used that send data over wireless connections to the processing unit. Wireless connections are, however, unreliable, often causing (partial) loss of data and performance degradation. Third, the deep neural network correctly estimated vGRF to be zero when the foot is airborne, which is a significant advantage over the non-zero vGRF measured by insoles when the foot moved towards the ground. Fourth, our system is not limited to estimating only vGRF and GCT, it also enables the estimation of most parameters relevant for gait analytics.

Ancillao et al. [4] discuss three further critical aspects of GRF estimation based on kinematic data: (1) how to determine the antero-posterior and medio-lateral components of GRFs, (2) how to determine GRF for each foot in double support conditions, and (3) how to estimate the absolute values of peak forces. All three problems could be solved using our system but were out of scope for this paper.

One open problem is to improve the presented deep neural networks such that they can also predict vGRFs and GCTs accurately for subjects for whom no training data are available. Therefore, collecting data from more test subjects is planned, and further network optimization will be carried out. Another limitation of the current version is that it has only been tested for walking and running on level terrain. In future research, other activities and types of terrains will be studied. Falbriard et al. [26] noticed in their study of foot-worn inertial sensors that the speed had a significant impact on the biases of gait parameter estimates. They therefore suggest a speed-dependent correction. Developing and testing such a correction method and analyzing potential biases in the gait parameter estimates is part of our future research.

## Data Availability

The three datasets used in this paper are available for download at http://urn.fi/urn:nbn:fi:att:4e6d3f54-1e87-4522-a6cf-cea979e6c236 (accessed on 18 January 2021).

## Abbreviations

The following abbreviations are used in this manuscript:

3D	Three-dimensional
4G	Fourth-generation broadband cellular network technology
BW	Bodyweight
GCT	Ground contact time
GCTL	Ground contact time of left foot
GCTR	Ground contact time of right foot
GPS	Global positioning system
GRF	3D Ground reaction force
GRU	Gated recurrent units
IMU	Inertial measurement unit
impulse_L	Left foot impulse during stride
impulse_R	Right foot impulse during stride
INS	Inertial navigation system
INS/GPS	GPS-aided inertial navigation system
*K*NN	*K* nearest neighbor
LSTM	Long short-term memory
LTE	Long term evolution
NRMSE	Normalized root mean square error
ReLU	Rectified linear unit
RMS	Root mean square
RNN	Recurrent neural network
TD	Touch-down event
TDL	Touch-down event left foot
TDR	Touch-down event right foot
TO	Toe-off event
TOL	Toe-off event left foot
TOR	Toe-off event right foot
USB	Universal serial bus
vGRF	Vertical ground reaction force
vGRFL	Vertical ground reaction force of left foot
vGRFR	Vertical ground reaction force of right foot
